# *Sclerocarya birrea* (Marula) Extract Inhibits Hepatic Steatosis in *db/db* Mice

**DOI:** 10.3390/ijerph19073782

**Published:** 2022-03-22

**Authors:** Lawrence Mabasa, Anri Kotze, Samukelisiwe Shabalala, Clare Kimani, Kwazi Gabuza, Rabia Johnson, Nonhlakanipho F. Sangweni, Vinesh Maharaj, Christo J. F. Muller

**Affiliations:** 1 Biomedical Research and Innovation Platform (BRIP), South African Medical Research Council (SAMRC), Tygerberg 7505, South Africa; anri.kotze@mrc.ac.za (A.K.); samukelisiwe.shabalala@mrc.ac.za (S.S.); clare.kimani@mrc.ac.za (C.K.); kwazi.gabuza@mrc.ac.za (K.G.); rabia.johnson@mrc.ac.za (R.J.); nonhlakanipho.sangweni@mrc.ac.za (N.F.S.); christo.muller@mrc.ac.za (C.J.F.M.); 2 Centre for Cardiometabolic Research in Africa, Division of Medical Physiology, Faculty of Medicine and Health Sciences, Stellenbosch University, Tygerberg 7505, South Africa; 3 Division of Medical Microbiology, Department of Pathology and Laboratory-Medicine, Faculty of Health Sciences, Walter Sisulu University, Mthatha 5117, South Africa; 4 Division of Clinical Pharmacology, Department of Medicine, Faculty of Medicine and Health Sciences, Stellenbosch University, Tygerberg 7505, South Africa; 5 Institute of Primate Research, P.O. Box 24481, Karen, Nairobi 00502, Kenya; 6 Department of Chemistry, University of Pretoria, Pretoria 0001, South Africa; vinesh.maharaj@up.ac.za; 7 Department of Biochemistry and Microbiology, University of Zululand, KwaDlangezwa 3880, South Africa

**Keywords:** Marula leaf extract, non-alcoholic fatty liver disease, DNA methylation, methylenetetrahydrofolate reductase, β-oxidation

## Abstract

Non-alcoholic fatty liver disease (NAFLD) is a spectrum of hepatic metabolic perturbations ranging from simple steatosis to steatohepatitis, cirrhosis and hepatocellular carcinoma. Currently, lifestyle modifications to reduce weight gain are considered the most effective means of preventing and treating the disease. The aim of the present study was to determine the therapeutic benefit of *Sclerocarya birrea* (Marula leaf extract, MLE) on hepatic steatosis. Obese *db/db* mice were randomly stratified into the obese control, metformin (MET) or MLE-treated groups. Mice were treated daily for 29 days, at which point all mice were euthanized and liver samples were collected. Hematoxylin and eosin staining was used for histological assessment of the liver sections, while qRT-PCR and Western blot were used to determine hepatic mRNA and protein expression, respectively. Thereafter, the association between methylenetetrahydrofolate reductase (Mthfr a key enzyme in one-carbon metabolism and DNA-methylation-induced regulation of gene transcription) and lipogenic genes was evaluated using Pearson’s correlation coefficient. Mice treated with MLE presented with significantly lower body and liver weights as compared with the obese control and MET-treated mice (*p* ≤ 0.05). Further, MLE treatment significantly inhibited hepatic steatosis as compared with the obese control and MET-treated mice (*p* ≤ 0.05). The reduced lipid accumulation was associated with low expression of fatty acid synthase (*Cpt1*; *p* ≤ 0.05) and an upregulation of the fatty acid oxidation gene, carnitine palmitoyltransferase (*Cpt1*; *p* ≤ 0.01), as compared with the obese control mice. Interestingly, MLE treatment improved the correlation between *Mthfr* and *Cpt1* mRNA expression (*r* = 0.72, *p* ≤ 0.01). Taken together, the results suggest that Marula leaf extracts may inhibit hepatic steatosis by influencing the association between *Mthfr* and genes involved in hepatic lipid metabolism. Further studies are warranted to assess DNA methylation changes in lipid metabolism genes.

## 1. Introduction

The global rise in the prevalence of obesity has brought about increases in a myriad of metabolic diseases, including type 2 diabetes mellitus (T2DM), non-alcoholic fatty liver disease (NAFLD) and cardiovascular diseases (CVD). Non-alcoholic fatty liver disease is a metabolic disorder characterized by an excess hepatic lipid buildup, with intrahepatic triglycerides accounting for more than 5% of the liver volume or weight [1]. It is a broad spectrum of liver disorders ranging from simple steatosis to non-alcoholic steatohepatitis, fibrosis and ultimately progressing to cirrhosis and hepatocellular carcinoma [2]. The metabolic spectra associated with NAFLD onset include increased adipose tissue lipolysis, increased hepatic lipid uptake and de novo lipogenesis with reduced beta-oxidation of fatty acids [3]. It has been reported that T2DM is associated with an increased risk of NAFLD, with two-thirds of T2DM presenting with NAFLD [4]. Type 2 diabetes mellitus and obesity is characterized by the body’s inability to effectively process circulating glucose due to peripheral insulin resistance (IR). It is postulated that the link between diabetes and NAFLD entails adipocyte-released pro-inflammatory cytokines conferring hepatic IR while promoting de novo lipogenesis and a reduction in β-oxidation of free fatty acids (FFAs). The result, therefore, is excessive hepatic lipid accumulation, a hallmark of steatosis. Currently, there is no FDA-approved treatment for NAFLD, with lifestyle interventions such as diet and exercise being recommended. In recent years, there has been a growing interest in plant-derived bioactive compounds and extracts due to their health benefits, with polyphenols such as those comprised in green tea shown to have beneficial effects against high fat diet-induced hepatic IR, inflammation and associated steatosis [5]. However, a better understanding of the disease pathophysiology is integral in developing preventative and therapeutic strategies. Indeed, there has been significant progress in our understanding of NAFLD, mainly through the utilization of multiple genome-wide association and larger candidate gene platforms [6]. To this end, there has been widespread interest in the role of epigenetics in gene transcription regulation, as well as transcriptomic profiling of the energy metabolism gene milieu and associated epigenetic marks associated with hepatic lipid accumulation. DNA methylation is one of the most studied epigenetic phenomena, which is known to influence heritable gene transcription without changes to the DNA sequence. It is influenced by the one-carbon metabolism pathway, which encompasses methionine and folate cycle reactions that either influence DNA synthesis or generate one-carbon units used in methylation reactions. Methylenetetrahydrofolate reductase (MTHFR) is one of the key enzymes involved in the pathway. It has been linked to increased disease susceptibility to NAFLD and has been proposed as a potential therapeutic target for various diseases [7]. Pertinent to this, of interest to this study is *Sclerocarya birrea* (*S. birrea*), commonly known as the Marula tree, of which the stem bark has traditionally been used as an antidiabetic remedy in sub-Saharan regions and Cameroon [8]. Thus, the aim of the present study was to determine the therapeutic benefit of Marula leaf extract (MLE) in hepatic steatosis and to ascertain a possible mechanistic link responsible for the outcome.

## 2. Materials and Methods

### 2.1. Sample Collection and Extraction

Marula leaves were collected by the Agricultural Research Council (ARC) in Mpumalanga, South Africa, with the extraction and spray drying of the aqueous extracts performed by the Council for Scientific and Industrial Research (CSIR) and the sample ID designed as VJM-I-55A. Extraction was performed with de-ionized water on dry-milled powder in a 50 L stainless steel electric stirrer running at slow speed. The slurry was transferred into a hydraulic press to separate the biomass from the liquid through a 50 micron filter bag at the highest pressure of 300 bar. The filtrate was spray-dried in a gas flow of 380 kg/h, with a rotary atomizer wheel speed of 2600 rpm, inlet temperature of 180 °C, outlet temperature of 80 °C, wall sweep temperature of 80 °C, wall sweep process gas and pneumatic hammer pressures of 1 bar and filter bag cleaning pressure of 5 bar.

### 2.2. Animals and Treatment

All animal procedures were conducted in accordance with the protocol approved by the South African Medical Research Council (SAMRC) Ethics Committee for Research on Animals (ECRA) (REF#06-19). Nine-week-old diabetic BKS.Cg-Dock7(m) +/+ Lepr(db)/J mice were kept in a temperature-controlled room at 22–25 °C, 45–55% humidity, with a 12 h light/dark cycle. After acclimation, 10-week-old mice were randomly assigned to either the control (obese C), metformin (obese + MET (500 mg/kg) (Glucophage 500 mg; Merck, New Jersey, USA) or aqueous Marula leaf extract (obese+ MLE (600 mg/kg)) groups. While water was used as a vehicle control, MET was used as a positive control (in the context of diabetes, a risk factor for NAFLD) and MLE as a test substance. Oral gavage was used as a mode of treatment administration. Body weight and non-fasted blood glucose were measured (using a glucometer via tail prick) once a week for the duration of the study. The mice were fed a standard rodent maintenance diet and their respective treatments for 29 days, at which point all mice were euthanized by exsanguination under isoflurane anesthesia. At termination, liver tissues were collected and either snap-frozen (stored at −80 °C), stored in RNALater (Ambion, Austin, TX, USA) or fixed in 4% buffered formaldehyde for subsequent assays.

### 2.3. Histology

For morphological changes and confirmation of lipid accumulation, formalin-fixed and paraffin-embedded liver sections were stained with hematoxylin and eosin (H&E) as previously described [9]. The H&E sections were evaluated by light microscopy and ImageJ v1.50i software was used for semi-quantitative analysis of hepatic lipid accumulation. Blinded histopathological assessment of H&E-stained liver sections was performed following the generic rodent steatosis analysis as suggested by Liang et al. [10].

### 2.4. mRNA Expression

#### 2.4.1. RNA Extraction and the Synthesis of Complimentary DNA

For the determination of differential gene transcription, total RNA was extracted from liver tissues (stored in RNALater) using a Qiagen Mini RNeasy kit according to the manufacturer’s instructions (Qiagen, Hilden, Germany). The quantity and quality of the total RNA was determined using a Nanodrop^®^ One spectrophotometer (ThermoFisher Scientific, Waltham, MA, USA) and 1.5% agarose gel, respectively, visualized in Bio-Rad Check MP System (Bio-Rad, Hercules, CA, USA). RNA samples were purified to eliminate genomic DNA using an Ambion Turbo DNase kit following the manufacturer’s instructions (Ambion, Austin, TX, USA). Thereafter, 1 µg of total RNA was used to synthesize a complimentary DNA sample using the High-Capacity cDNA Reverse Transcription Kit as per the manufacturer’s protocol (Applied Biosystems, Waltham, MA, USA).

#### 2.4.2. Quantitative Real-Time Polymerase Chain Reaction (qRT-PCR)

For analyses of gene transcription, qRT-PCR was performed for amplification of cDNA using 2x FastStart TaqMan^®^ PCR Master Mix and TaqMan assays (ThermoFisher Scientific, Waltham, MA, USA) under the following cycling conditions: pre-incubated at 95 °C for 5 min followed by 40 cycles of denaturation at 95 °C for 10 s, annealing at 60 °C for 10 s and extension at 70 °C for 10 s on a QuantStudio^TM^ 7 Flex Real-Time PCR system (ThermoFisher Scientific, Waltham, MA, USA). The Taqman assays covered genes involved in insulin signaling (protein kinase B (*Akt*), phosphatidyl-inositol-3-kinases (*PI3k*), insulin receptor substrate 2 (*Irs2*), glucose transporter 2 (*Glut2*)), β-oxidation (peroxisome proliferator-activated receptor alpha (*Pparα*), AMP-activated protein kinase (*Ampk*), carnitine palmitoyltransferase 1 (*Cpt1*)), lipogenesis (acetyl-CoA carboxylase (*Acc*), sterol regulatory element binding transcription factor 1 (*Srebf1*), fatty acid synthase (Fasn)), one-carbon metabolism or DNA methylation (DNA methyltransferase 1 (*Dnmt1*) and (methylenetetrahydrofolate reductase (*Mthfr*)) and trans-sulfuration (cystathionine β-reductase (*Cbs*)). Beta-actin (*ActB*) and hypoxanthine phosphoribosyl transferase 1 (*Hprt1*) were used as housekeeping genes. A standard curve-based method was used for data processing.

### 2.5. Protein Expression

#### 2.5.1. Protein Extraction

A gene shown to be differentially expressed (FASN) was validated to assess the protein level by Western blot experiment. Total protein extraction was conducted as previously described in [11]. Briefly, the total protein was extracted from 25.0 to 30.0 g of frozen liver tissue using a chilled lysis buffer (Pierce Biotechnologies, Rockford, CA, USA). The tissue lysate was then centrifuged before the supernatant was collected. Thereafter, protein quantification was conducted by using the Bio-Rad DC Protein Assay kit following the manufacturer’s protocol (Bio-Rad, Hercules, CA, USA).

#### 2.5.2. Western Blotting

Thirty micrograms of protein lysate was denatured at 95 degrees and loaded onto 4 or 6% SDS gel (Bio-Rad, Hercules, CA, USA) for the separation of proteins. Following separation by gel electrophoresis at 120 V for around 70 min, proteins were transferred to a polyvinylidene fluoride (PVDF) membrane (Bio-Rad, Hercules, CA, USA) using the Trans-Blot Turbo Transfer System at 25 V for 10 min. Thereafter, the membranes were incubated in 5% non-fat skimmed milk (Sigma-Aldrich, St. Louis, MI, USA) in Tris-buffered saline with Tween-20 (TBST) at room temperature for 2 h to block non-specific proteins. The membrane was then incubated in a primary antibody, anti-FASN, at 1:1000 (Cell Signaling, Danvers, MA, USA) overnight at 4 °C. Following the overnight incubation, the membranes were washed 3 times and incubated in a secondary antibody (anti-rabbit IgG-HRP,1:4000) conjugated with relevant horseradish peroxidase at room temperature for a total of 90 min. After the secondary antibody incubation, the membranes were rinsed 3 times before proteins were detected and quantified using ChemiDoc MP system (Bio-Rad, Hercules, CA, USA). Proteins were normalized to beta-actin (1:500) (Santa Cruz Biotechnology, CA, USA), which was used as a loading control.

### 2.6. Statistical Analysis

Data were analyzed using GraphPad Prism software versions 6 and 9 (for validation) (California, CA, USA) and are expressed as means ± standard errors of the mean (SEM). In cases where data were found to be normally distributed with equal variance, parametric tests such as ANOVA or *t*-tests were used. Statistical significance was considered as *p* ≤ 0.05. Further, for correlations between *Mthfr* and genes involved in hepatic lipid metabolism, Pearson’s correlation coefficient was computed [12].

## 3. Results

### 3.1. Bodyweight and Non-Fasted Blood Glucose

**Figure 1:**
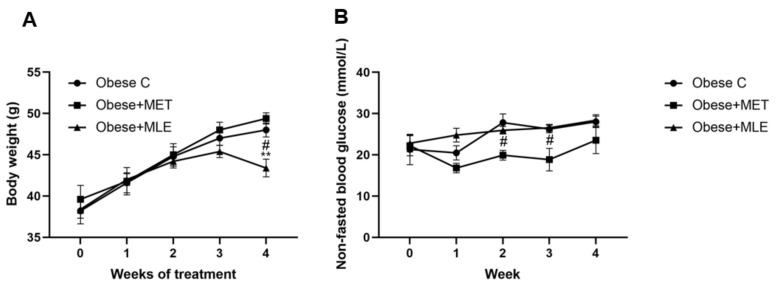
Bodyweight (**A**) and non-fasted blood glucose (**B**) results for *db/db* mice. Data are represented as means ± SEM, with a sample size of 5 animals/group. GraphPad Prism 6 was used to test for statistical significance at a *p*-value ≤ 0.05.

After 4 weeks of treatment, obese + MLE mice presented with significantly lower bodyweight as compared with the obese C (*p* < 0.05) and obese + MET (*p* < 0.01) mice (Figure 1A). It is worth noting that the difference became evident at 3 weeks, although this was not statistically significant. Further, as expected, obese + MET mice showed significant reductions in non-fasted blood glucose at 2 and 3 weeks of treatment as compared to both obese C and obese + MLE (*p* < 0.05) groups, while MLE did not affect blood glucose as compared with the obese C mice (Figure 1B). The difference observed became apparent after 1 week of treatment, although this was not statistically significant.

### 3.2. Liver Weight and Lipid Accumulation

**Figure 2:**
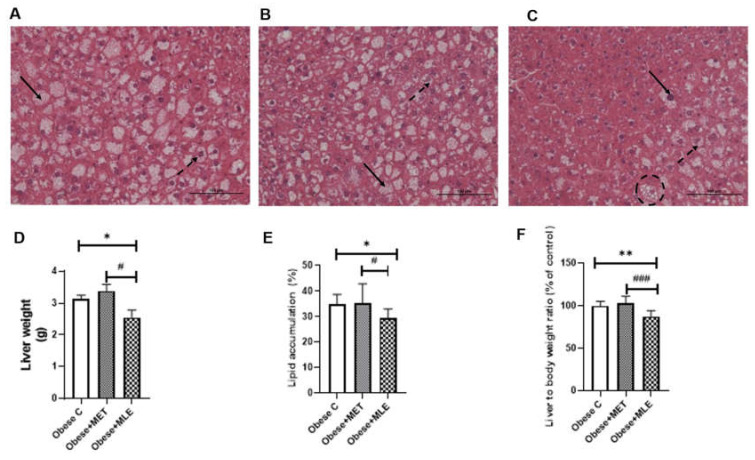
Hepatocellular steatosis, liver weight and percent lipid accumulation results. Representative photomicrographs of liver cross-sections of the obese C (**A**), obese + MET (**B**) and obese + MLE (**C**) mice. Histologically steatosis severity was classified by the number and distribution of hypertrophied hepatocytes (dotted circle) with either macrovesicular (bold arrows) or microvesicular (dotted arrows) steatosis. All photomicrographs were stained with H&E at 40× magnification. Liver weight (D), % lipid accumulation (E), and liver to body weight ratio (F) results are represented as means ± SEM, with a sample size of 5/group (10 images per slide were taken). GraphPad Prism 9 was used to test for statistical significance at a *p*-value ≤ 0.05. Note: * *p* ≤ 0.05 and ** *p* ≤ 001 vs. obese C, # *p* ≤ 0.05 and ### *p* ≤ 0.0001 vs. obese + MET.

The results obtained showed that mean liver weights and liver weight to body weight ratios were significantly lower in obese + MLE mice as compared with the obese C and obese + MET mice (Figure 2D (*p* ≤ 0.05) and Figure 2F (*p* ≤ 0.01 and *p* ≤ 0.001, respectively)), while no differences were observed between MET-treated mice and the obese C group. This was further supported by histology data on hepatic lipid accumulation, with the obese C (Figure 2A,E) and obese + MET (Figure 2B,E) groups displaying microvesicular steatosis, as identified by the accumulation of multiple small lipid droplets in hepatocytes. The obese + MLE group showed reductions in the area and severity of steatosis.

### 3.3. Gene Transcription and Translation

#### 3.3.1. De Novo Lipogenesis

**Figure 3:**
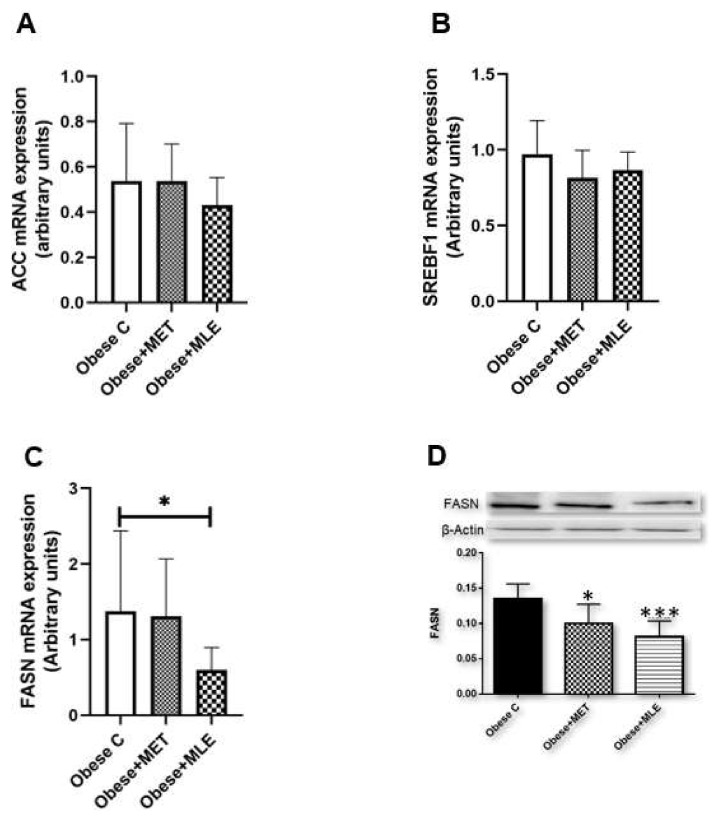
The effects of Marula leaf extract (MLE) on mRNA expression of lipogenic genes, *Acc* (**A**), *Srebf1* (**B**), and *Fasn* (**C**), as well as protein levels of FASN (**D**). Data are represented as means ± SEM, with a sample size of 5–6/group. GraphPad Prism 9 was used to test for statistical significance at a *p*-value ≤ 0.05. Note: * *p* ≤ 0.05 vs. obese C, *** *p* ≤ 0.001 vs. obese C. *Acc*; acetyl-CoA carboxylase, *Srebf1*; sterol regulatory element binding transcription factor 1, *Fasn*; fatty acid synthase.

Hepatic lipid accumulation is dependent on the uptake of circulating plasma fatty acids as well as de novo lipogenesis (DNL), the latter of which is largely transcriptionally regulated. Thus, the study sought to ascertain the influence of Marula leaf extract (MLE) on the gene regulatory network associated with DNL. While MLE did not significantly affect mRNA expression of acetyl-CoA carboxylase (*Acc*) or sterol regulatory element binding transcription factor 1 (*Srebf1*) (Figure 3A,B), mice presented with a significant reduction (Figure 3C; *p* ≤ 0.05) in fatty acid synthase (*Fasn*), the rate-limiting enzyme in DNL, as compared with the obese C group. These findings were further validated by Western blot data, where FASN protein expression was significantly reduced by MLE treatment as compared with the obese C group (Figure 3D; *p* < 0.05). Interestingly, metformin (MET) treatment significantly reduced FASN levels as compared with the obese C group (*p* < 0.05).

#### 3.3.2. β-Oxidation

**Figure 4:**
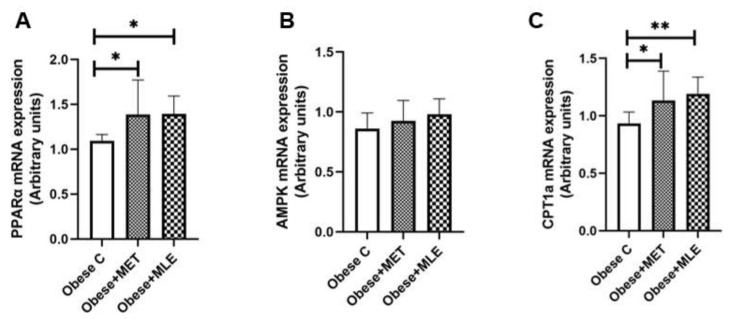
The effects of Marula leaf extract (MLE) on mRNA expression of β-oxidation genes, *Pparα* (**A**), *Ampk* (**B**) and *Cpt1* (**C**). Data are represented as means ± SEM, with a sample size of 5-6/group. One-way ANOVA was used to test for statistical significance at a *p*-value ≤ 0.05. Note: * *p* ≤ 0.05 vs. obese C, ** *p* ≤ 0.01 vs. obese C. *Pparα*; peroxisome proliferator-activated receptor alpha, Ampk; AMP-activated protein kinase, Cpt1; carnitine palmitoyltransferase I.

Hepatic steatosis, a hallmark of non-alcoholic fatty liver disease (NAFLD), arises when there is an imbalance between lipid acquisition (i.e., fatty acid uptake and lipogenesis) and expenditure (i.e., lipid export and β-oxidation). Thus, we assessed the role of MLE on mRNA expression of key β-oxidation genes, peroxisome-proliferator-activated receptor alpha (*Pparα*), AMP-activated protein kinase (*Ampk*) and carnitine palmitoyltransferase I (*Cpt1*). Here, obese + MET mice presented with significant increases in *Pparα* and *Cpt1* (Figure 4A,C; *p* < 0.05), while *Ampk* mRNA expression (Figure 4B) was not affected as compared with the obese C group. Of particular interest, the data revealed that obese + MLE mice had significantly higher levels of *Pparα* (Figure 4A; *p* < 0.05) and *Cpt1* (Figure 4C; *p* < 0.01), while *Ampk* was not affected as compared with the obese C group (Figure 4B). Of the 3 genes studied, we did not observe any differences between the obese + MLE and obese + MET mice.

#### 3.3.3. Insulin Signaling

**Figure 5:**
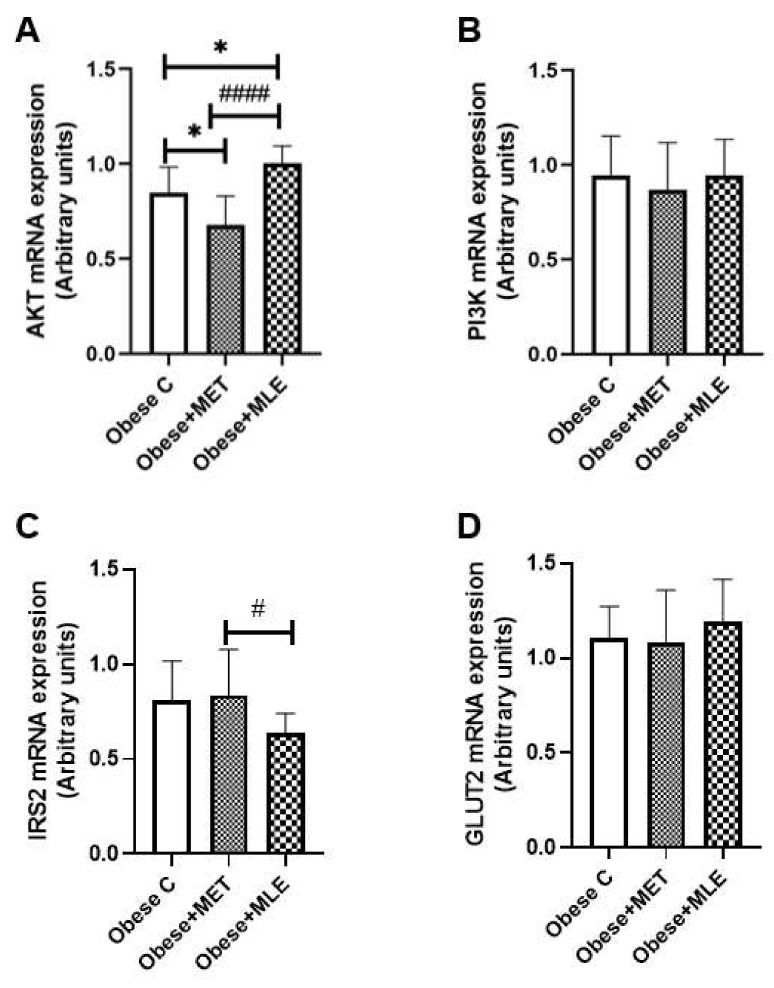
The effects of Marula leaf extract (MLE) on mRNA expression of insulin signaling genes, *Akt* (**A**), *Pi3k* (**B**), *Irs2* (**C**) and *Glut2* (**D**). Data are represented as means ± SEM, with a sample size of 5-6/group. GraphPad Prism 9 was used to test for statistical significance at a *p*-value ≤ 0.05. Note: * *p* ≤ 0.05 vs. obese C, # *p* ≤ 0.05 vs. obese + MET and #### *p* ≤ 0.0001 vs. obese + MET. *Akt*; protein kinase B, *PI3k*; phosphatidyl-inositol-3-kinases, *Irs2*; insulin receptor substrate 2, *Glut2*; glucose transporter 2.

Non-alcoholic fatty liver disease commonly occurs in the background of insulin resistance, a hallmark of T2DM. As such, this study aimed to determine the effects of MLE on genes involved in insulin signaling, namely Akt, phosphatidyl-inositol-3-kinases (PI3k), Irs2 and Glut2. The results showed that obese + MET mice had significantly reduced hepatic Akt mRNA levels as compared with the obese C group (Figure 5A; *p* < 0.05). Further, treatment with MLE induced a significant increase in the expression of Akt mRNA when compared with both the obese + MET and obese C mice (*p* < 0.0001 and *p* < 0.05, respectively). In addition, while obese C and obese + MET groups did not differ in terms of the Irs2 mRNA expression levels, MLE-treated mice presented with a significantly lower expression level of Irs2 only when compared with obese + MET mice (Figure 5C; *p* < 0.05). However, neither treatment affected the mRNA expression levels of PI3k (Figure 5B) or Glut2 (Figure 5D) as compared with the obese C mice.

#### 3.3.4. One-Carbon Metabolism and DNA Methylation

**Figure 6:**
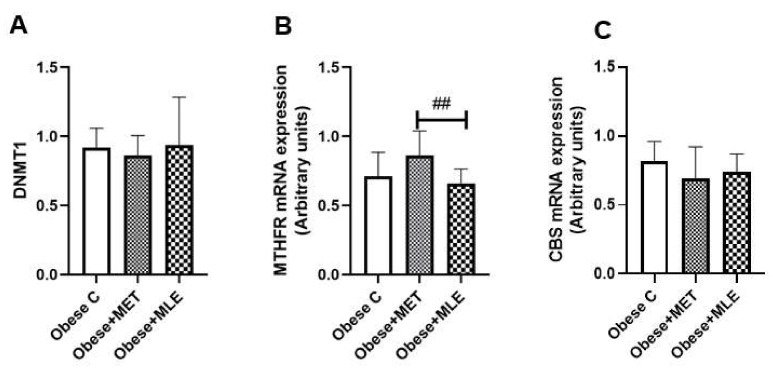
The effects of Marula leaf extract (MLE) on mRNA expression *Dnmt1* (**A**), *Mthfr* (**B**) and *Cbs* (**C**) levels of genes involved in the control of DNA methylation. Data are represented as means ± SEM, with a sample size of 5-6/group. GraphPad Prism 9 was used to test for statistical significance at a *p*-value ≤ 0.05. Note: ## *p* ≤ 0.01 vs. obese + MET. *Dnmt1*; DNA methyltransferase 1, *Mthfr*; methylene tetrahydrofolate reductase, *Cbs*; cystathionine-β-synthase.

One-carbon metabolism is a series of enzymatic reactions that function to generate methyl groups that are utilized in DNA methylation reactions. It encompasses folate and methionine cycles, while it is also closely associated with pathways such as transsulfuration. *Mthfr* mRNA expression was significantly higher in obese + MET mice as compared with obese-MLE mice (Figure 6B; *p* < 0.01). Though not significant, the obese + MET group displayed increased *Mthfr* mRNA expression as compared to the obese C group. Obese + MLE treatment did not influence the transcription of *Mthfr* when compared with the obese C mice. Neither treatment affected the expression levels of DNA methyltransferase 1 (*dnmt1*; Figure 6A) or cystathionine-β-synthase (*Cbs*; Figure 6C) as compared with the obese C mice.

#### 3.3.5. Association between MTHFR and Lipid Metabolism Gene Milieu

Here, we attempted to unravel the association between Mthfr and genes involved in hepatic lipid metabolism and to ascertain whether the inhibitory effect of MLE on hepatic steatosis could be explained by its ability to significantly influence these associations. To accomplish this, Pearson’s correlation coefficient was used. First, the association between Mthfr and hepatic lipid metabolism genes in non-treated obese diabetic *db/db* mice was established. As shown in Table 1, significant positive associations between Mthfr and the lipogenic genes Acc and Fasn (*r* = 0.67 and 0.71, respectively; *p* < 0.05) were recorded, while an inverse relationship with Ampk (*r* = −0.72; *p* < 0.05), a key regulator of lipid metabolism, was shown. Interestingly, MET treatment significantly reversed the associations of Mthfr with Acc and Fasn (*r* = −0.92 and −0.91, respectively; *p* ≤ 0.001). Interestingly, the data showed that MLE treatment significantly improved the correlation between Mthfr and the β-oxidation gene Cpt1 (*r* = 0.72; *p* < 0.01). No further significant associations were recorded, although obese + MET treatment tended to inversely affect the association between Mthfr and Akt, while obese + MLE treatment tended to inversely affect the association between Mthfr and Pparα.

## 4. Discussion

Non-alcoholic fatty liver disease (NAFLD) is a consequence of hepatic lipid metabolic perturbations characterized by hepatic steatosis (excess accumulation of fat). It ranges from simple steatosis to non-alcoholic steatohepatitis, fibrosis and ultimately progressing to cirrhosis and hepatocellular carcinoma. It is estimated that 1 in 4 people have NAFLD and this prevalence is expected to increase due to the rise in key risk factors such as obesity [13]. Further, it is reported that approximately 22% of adults living with T2DM have NAFLD [14]. Currently, lifestyle modifications to reduce weight gain are considered the most effective means of preventing and treating the disease. Nonetheless, in recent years, there has been a growing interest in plant-derived phenolic compounds and extracts due to their therapeutic potential, safety and availability. Indeed, phenolic compounds such as resveratrol have previously been shown to attenuate high fat diet-induced expression of the lipogenic genes *Fasn* and *Srebf1*, as well as related serum triglyceride levels [15].

Of interest to this study is *Sclerocarya birrea* (*S. birrea*), commonly known as the Marula tree, of which the stem bark has traditionally been used as an antidiabetic remedy in sub-Saharan regions and Cameroon [8]. Indeed, evidence has shown that *S. birrea* has hypoglycemic effect, in part via the potentiation of insulin secretion and glucose metabolism both in vitro and in rat animal models [16]. Further, the extract has been reported to lower plasma cholesterol and triglyceride levels in streptozotocin diabetic rats, highlighting its potential to influence lipid metabolism [17]. While the stem bark has been widely used, stripping of the Marula tree to harvest the stem bark is considered unsustainable; hence, studies have shifted the focus to Marula leaf extract. Indeed, profiling of *S. birrea* stem bark and leaf extracts revealed that they both have strong antioxidant properties and high polyphenolic content [18]. As such, the current study sought to assess the role of Marula leaf extract in hepatic steatosis and to ascertain whether its inhibitory effect on hepatic lipid accumulation may be due to its modulation of lipid metabolism signatures.

In this study, diabetic mice were grouped into obese C, obese + MET and obese + MLE groups. Mice were administered their respective treatments daily for 29 days, during which bodyweight and non-fasted blood glucose were measured weekly.

Obese + MLE mice presented with significantly lower bodyweight as compared with the obese C and obese + MET groups. This was in contrast with previous findings that showed that Marula stem bark extract did not affect bodyweight in streptozotocin-induced diabetic rats [17]. To ascertain whether the loss in bodyweight may have translated to the amelioration of hepatic steatosis, histology of H&E-stained liver sections was used to assess steatosis changes. As anticipated, we observed that obese + MLE mice had significantly smaller livers and less lipid droplets as compared with the obese + MET and obese C groups. According to our knowledge, this is the first demonstration of the hepatic lipid inhibitory effect of Marula leaf extract. Nonetheless, the results are in partial consonance with findings reported previously, where Marula fruit juice and stem bark extract attenuated plasma lipid profiles [17,19]. Profiling of phytochemicals in tissues from *S. birrea* using HPLC-MS revealed the presence of phenolic compounds such as epigallocatechin-3-gallate (EGCG) and myricetin [18]. While EGCG has previously been shown to attenuate body and liver weights and hepatic steatosis, myricetin has been reported to reduce serum triglycerides, insulin insensitivity and hepatic steatosis [20,21].

In the present study, we did not find a significant difference between the obese C and obese + MET groups in terms of hepatic steatosis. This is consistent with the findings of Min et al. [22], who showed that MET has no effect on high fat diet-induced hepatic lipid accumulation in mice. However, using C57BI/6J mice, a study by Brandt et al. [23] recently showed that MET treatment attenuates the onset of fat-, fructose- and cholesterol-rich diet-induced NAFLD, inflammation and lipid peroxidation. While Min et al. [22] used a lower dose of 200 mg/kg bodyweight, Brandt and colleagues [23] used 300 mg/kg bodyweight, both of which were lower than what was used in the current study (500 mg/kg bodyweight). However, the current study treated mice for 4 weeks, while Brandt et al. [23] used a 6 week treatment period, which could explain why MET was shown to be beneficial in the latter project. Further, the current study used *db/db* mice, which present with a severe form of obesity and hyperphagia due to a mutation on the leptin receptor, while the above studies used diet-induced disease models [24]. Indeed, Zhu et al. [25] previously reported that at 250 mg/kg bodyweight, MET treatment did not affect hepatic lipid levels in *db/db* mice as compared with the *db/db* vehicle control mice.

Further, the current study attempted to ascertain whether the inhibitory effect of MLE on the formation of hepatic lipid droplets could be due to the modulation of genes involved in lipid metabolism. Here, we showed that MLE treatment significantly downregulated the expression of *Fasn* while upregulating *Pparα* and *Cpt1*. While FASN is a terminal enzyme in de novo lipogenesis (DNL), *Pparα* is known to activate *Cpt1*, a key protein in β-oxidation. In NAFLD, DNL and fatty acid uptake exceeds the ability of the liver to prevent lipid build-up via β-oxidation and the export of very low-density lipoproteins [26]. Thus, data suggest that Marula leaf extract may suppress the accumulation of hepatic lipid droplets, in part via the activation of β-oxidation.

As indicated earlier, about one-third of T2DM patients have NAFLD, and this could be due to several factor, as follows: (1) insulin resistance (IR) impairing the ability of insulin to suppress adipose tissue lipolysis, thereby resulting in excess hepatic uptake of free fatty acids; (2) T2DM could be a consequence of NAFLD due to changes in hepatokine secretion that favor the induction of inflammation and IR in peripheral tissues [27]. In the current study, MLE did not affect non-fasted blood glucose, while as expected MET treatment significantly reduced blood glucose as compared with the obese C group. The results for MLE are not supported by previous studies that highlighted the antidiabetic properties of Marula extracts [16,17]. As a result, further studies are warranted, perhaps looking at different dosages and timelines, particularly since mRNA expression data revealed that obese + MLE treatment significantly upregulated the expression of protein kinase B (*Akt*) mRNA as compared with obese + MET and obese C groups. Interestingly, obese + MET treatment significantly reduced the expression of *Akt* as compared with the obese C group. In the insulin signaling pathway, *Akt* plays a crucial role by promoting glucose uptake, glycogen synthesis and lipid breakdown. The results for MET’s inhibitory effect on *Akt* mRNA are consistent with previous studies that showed that MET treatment suppresses the PI3K/AKT signaling in both cancer and NAFLD patients [28,29]. It could be argued that by modulating *Akt* kinase activity, perhaps MLE can reduce DNL while enhancing β-oxidation, thereby attenuating hepatic lipid accumulation. These dynamics require further investigations, particularly with epigenetic modulation of *Akt* gene transcription due to MLE treatment. Nonetheless, it is evident that while MET may work via an insulin-independent pathway, MLE uses the insulin-dependent pathway to exert its effect.

To further elucidate the detailed mechanism through which MLE regulated gene transcription in association with reduced hepatic steatosis, we sought to unravel the status of methylenetetrahydrofolate reductase (MTHFR), a key protein in one-carbon metabolism. Recently, mutations associated with MTHFR have been identified and linked with the susceptibility to developing an array of health complications. Indeed, mutations such as the MTHFR gene C677T have been closely associated with congenital heart disease and other cardiovascular disease risk factors, including hyperhomocysteinemia, hyperlipidemia and blood pressure [30,31,32,33]. Interestingly, NAFLD patients were previously shown to present with increased plasma homocysteine (Hcy), along with MTHFR C677T, when compared with healthy controls, further highlighting the involvement of MTHFR in the disease onset and progression [31,34].

The one-carbon metabolism pathway involves a series of metabolic reactions that function to maintain amino acid homeostasis while directly impacting epigenetic processes by generating methyl groups used in DNA methylation reactions. In the last decades, DNA methylation has been linked with the regulation of metabolic and health outcomes, in part due to its ability to affect the activity of genes involved in cellular processes. DNA methylation within the promoter region is believed to confer a repressive role on gene transcription, whereas methylation in the gene body is predominantly linked with upregulated gene transcription [35]. Methylenetetrahydrofolate reductase (MTHFR) is a rate-limiting enzyme in one-carbon metabolism, a pathway that affects DNA methylation reactions. Its role is to facilitate oxidation of 5,10-methylene tetrahydrofolate (5,10-methylene THF) to 5-methyl THF. The latter serves as a methyl donor in the conversion of Hcy to methionine, a process that requires the involvement of methionine synthase and vitamin B_12_ as a cofactor. Methionine is in turn utilized in the generation of s-adenosylmethionine, a critical methyl donor in DNA methylation reactions. Although the data presented here suggest that MLE may influence hepatic lipid metabolism via other mechanisms rather than through the modulation of *Mthfr* mRNA expression, it is the view of these researchers that perhaps the corresponding background levels of *Mthfr* in association with lipid metabolism genes are more relevant than the expression levels in isolation. Thus, we further performed a Pearson correlation analysis to determine the association between *Mthfr* and lipid metabolism genes and to ascertain whether MLE can influence this correlation in association with reduced hepatic steatosis. Indeed, in this study, MLE treatment significantly induced a positive association between *Mthfr* and *Cpt1*, a gene involved in β-oxidation.

**Figure 7:**
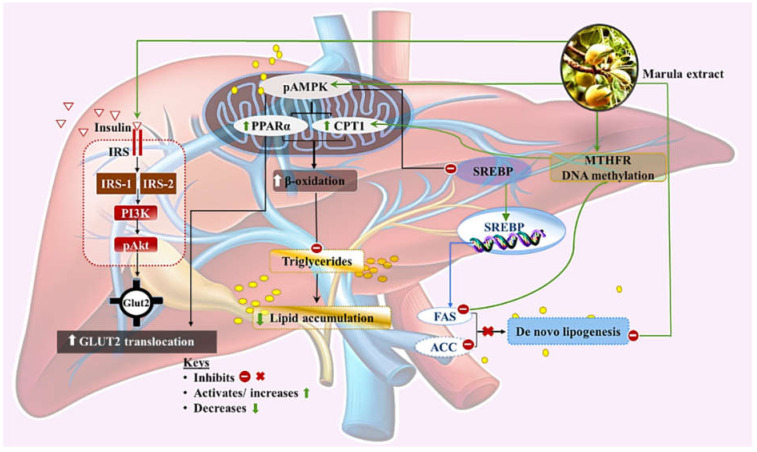
Schematic representation of the potential mechanism through which Marula leaf extract lowers hepatic lipid accumulation. Marula leaf extract can significantly influence the association between MTHFR and CPT1, which impacts the DNA methylation profile of Cpt1, and in turn triggers the activation of the β-oxidation pathway.

The working hypothesis (Figure 7) is that Marula leaf extract attenuates hepatic steatosis perhaps by impacting the epigenetic regulation of fatty acid oxidation (via the modulation of the association between *Mthfr* and *Cpt1*) and lipogenesis (through the repression of *Fasn* activity) genomic machineries; however, this link requires further investigation. Indeed, polyphenols such as catechins and myricetin, both of which have been identified in Marula leaf extract, have previously been shown to induce hypermethylation in the liver [18,36]. Alternatively, Marula leaf extract may impact the epigenetic regulation of *Pparα*, resulting in its upregulation, and subsequently the activation of *Cpt1* and fatty acid oxidation.

## 5. Conclusions

This study is the first to demonstrate the inhibitory effect of Marula leaf extract on hepatic steatosis in diabetic mice. This consequence appears to be modulated via the activation of β-oxidation and a reduction in lipogenesis, in part due to the regulation of the association between *Mthfr* and genes involved in lipid metabolism. Further studies are warranted to explore DNA methylation changes in lipid metabolism genes. While the presented here data are exciting, the study did have some limitations, including its small sample size, lack of a non-obese control and less tissue samples to perform protein expression analyses beyond FASN and DNA methylation analyses of lipid metabolism genes. Further, except for the limited biological specimens, the quantification of hepatic lipids using Oil Red O and assessment of liver function using blood markers such as transaminases would have been more informative. In addition, the study made use of a genetic model of obesity and diabetes, which may differ from the human phenotype [37]. However, the homozygous diabetic leptin-resistant BKS.Cg-Dock7m+/+ Leprdb/J (*db/db*) mice model used here has been a useful tool in studies of liver dysfunction, as the animals have previously been shown to exhibit hepatic steatosis and lobular inflammation, along with significantly elevated alanine transaminase, aspartate aminotransferase, total cholesterol and triglyceride levels as compared with non-diabetic mice [38,39]. To address several of the highlighted limitations, the use of a high-fat, high-cholesterol diet model of hepatic steatosis in rodents may be beneficial. Nonetheless, the data presented here may serve as a great platform for future studies, as they demonstrate strong evidence that Marula leaf extract has the potency to inhibit hepatic steatosis.

## Figures and Tables

**Table 1:** Pearson’s correlation analysis of hepatic methylenetetrahydrofolate reductase (MTHFR) with genes involved in insulin signaling, beta-oxidation and DNA methylation.

Pearson Correlation Coefficient											
	Genes (r (*p*-Value))										
Groups	*Acc*	*Ppar*	*Fasn*	*Cpt1*	*Ampk*	*Srebf1*	*Irs1*	*Akt*	*Glut2*	*PI3k*	*Cbs*
obese C	0.67 (0.03)	0.55 (0.10)	0.71 (0.02)	−0.42 (0.23)	−0.72 (0.02)	0.58 (0.08)	−0.09 (0.81)	0.53 (0.12)	0.04 (0.91)	0.59 (0.08)	−0.78 (0.01)
obese + MET	−0.92 (0.001)	−0.55 (0.10)	−0.91 (0.001)	0.45 (0.20)	−0.49 (0.15)	−0.26 (0.46)	0 (1.0)	−0.61 (0.06)	−0.31 (0.38)	−0.42 (0.23)	−0.14 (0.69)
obese + MLE	−0.09 (0.76)	−0.51 (0.06)	0.32 (0.27)	0.72 (0.003)	−0.09 (0.77)	0.31 (0.28)	−0.27 (0.35)	0.44 (0.11)	0.20 (0.49)	−0.28 (0.33)	0.38 (0.18)

Mthfr: methylenetetrahydrofolate reductase, MET: metformin, MLE: Marula leaf extract, acc; acetyl-CoA carboxylase, Srebf1; sterol regulatory element binding transcription factor 1, Fasn; fatty acid synthase, Pparα; peroxisome proliferator-activated receptor alpha, Ampk; AMP-activated protein kinase, Cpt1; carnitine palmitoyltransferase I, AKT; protein kinase B, PI3k; phosphatidyl-inositol-3-kinases, Irs2; insulin receptor substrate 2, Glut2; glucose transporter 2, Dnmt1; DNA methyltransferase 1, Mthfr; methylene tetrahydrofolate reductase, Cbs; cystathionine-β-synthase.

## Data Availability

Data reported from this study is contained within the article or supplemental material (where applicable).
